# Risk assessment of low-temperature biochar used as soil amendment on soil mesofauna

**DOI:** 10.1007/s11356-019-05153-7

**Published:** 2019-04-30

**Authors:** Iwona Gruss, Jacek P. Twardowski, Agnieszka Latawiec, Agnieszka Medyńska-Juraszek, Jolanta Królczyk

**Affiliations:** 1 Department of Plant Protection, Wroclaw University of Environmental and Life Sciences, Grunwaldzka 24a, 50-363 Wrocław, Poland; 2 Institute of Agricultural Engineering and Informatics, University of Agriculture in Kraków, Balicka 116B, 30-149 Kraków, Poland; 3 Department of Geography and the Environment, Rio Conservation and Sustainability Science Centre, Pontifícia Universidade Católica, Rio de Janeiro, 22453-900 Brazil; 4 Institute of Soil Sciences and Environmental Protection, Wroclaw University of Environmental and Life Sciences, Grunwaldzka 53, 50-357 Wrocław, Poland; 5 Department of Manufacturing Engineering and Production Automation, Faculty of Mechanical Engineering, Opole University of Technology, Mikołajczyka 5, 45-271 Opole, Poland

**Keywords:** Biochar, Springtails, Mites, Soil quality, Avoidance, Reproduction

## Abstract

Biochar as a carbon-rich highly porous substance has been proposed for use in agriculture and horticulture as a soil amendment. One of the main concerns of this application of biochar is its potential contamination with heavy metals (HMs) and polycyclic aromatic hydrocarbons. The aim of this research was to access the environmental risk of biochar used as a soil amendment on soil mesofauna (mites and springtails). We conducted both field and laboratory experiments with the use of wood-chip biochar from low-temperature (300 °C) flash pyrolysis. Biochar was free from polycyclic aromatic hydrocarbons (PAH), and the concentration of all tested toxic compounds was very low or even under the level of detection. Both the results of field and laboratory studies show no toxic effects on soil mesofauna. In the field studies, the biochar application of 50 t/ha in maize and oilseed rape crops significantly increased the mean number of mesofauna. This change probably resulted from improved soil chemical properties (in particular organic carbon content and cation exchange capacity) upon biochar addition. The results of the avoidance test with the use of springtail species *Folsomia candida* showed the possible short-term toxicity risk from a dose of 5%. The results of the reproduction test indicate the negative response of *F. candida* from the rate of 25% (higher than the field dose, which corresponds to 10% in laboratory tests). The reason for the short-term toxicity might be the considerable increase in soil pH after biochar addition. To our knowledge, this is the first study that has looked so widely into the effect of biochar on soil mesofauna. We encourage further studies into the risk assessment of biochar on soil organisms in both a controlled laboratory environment and in the open field.

## Introduction

Biochar is one of the products of biomass gasification in a certain range of temperatures and with limited oxygen access, also called pyrolysis. It is a solid, carbon-rich, stable, and highly recalcitrant residue (Hansen et al. 2015). Due to the specific physical and chemical nature of biochar, it has been proposed as a soil amendment to improve soil bulk density, soil pH of acid soils, to increase the stability of organic soil’s matter content, and to improve nutrient retention through cation adsorption (Lehmann et al. 2006; Liang et al. 2006; Domene et al. 2014; Marks et al. 2014; Latawiec et al. 2017b). Biochar has also been shown to improve soil aggregate stability and water availability (Ma et al. 2016; Zhang et al. 2017). Knowledge about the consequences of the use of biochar on the environment in general is, however, scarce (Kuppusamy et al. 2016; Latawiec et al. 2017a). One of the main concerns of biochar use as a soil amendment is its potential contamination with heavy metals (HMs) and polycyclicaromatic hydrocarbons (PAH) (Freddo et al. 2012). Pyrolysis technologies have been shown to increase the potential pollutant concentration of the original feedstock due to mass losses (Méndez et al. 2012; Farrell et al. 2013). Generally, the heavy metal content and alkalify of biochar can increase with the pyrolysis temperature (Chen et al. 2014). However, heavy metals are also characteristic for biochars from low-temperature pyrolysis (Domene et al. 2015a; b). Additionally, temperatures of pyrolysis up to 500 °C can result in maximum concentrations of PAH in biochar (Keiluweit et al. 2012). In the study on the effects of pyrolysis temperature on the potential toxicity of biochar, PAH were mostly produced at temperatures of 300–400 °C (Lyu et al. 2016). Also, different kinds of dioxins were found in biochars produced at temperatures from 250 to 900 °C (Hale et al. 2012).

An important aspect related to the use of biochar in agricultural crops is its effect on soil biota and ecosystem services (McCormack et al. 2013). Positive effects of biochar on microbial abundance and activity have been reported in many studies (Pietikäinen et al. 2000; Steiner et al. 2004; Birk et al. 2009; Zhang et al. 2017). Conversely, the changes in microbial community composition may affect nutrient cycles, plant growth, and the cycling of organic soil matter (Kuzyakov et al. 2009; Liang et al. 2010). The interaction between biochar and earthworm abundance and activity is also relatively well studied in field trials (Topoliantz and Ponge 2005; Tammeorg et al. 2014) and in laboratory tests (Liesch et al. 2010; Li et al. 2011; Malińska et al. 2016; Malińska et al. 2017). The response of other groups of soil biota to biochar is far less studied. Soil mesofauna are mostly represented by springtails (Hexapoda: Collembola) and soil mites (Arachnida: Acari), accounting for about 95% of the total number of arthropods inhabiting soil (Neher and Barbercheck 1998). Most mesofauna representatives live mostly in the surface soil layer, down to 10 cm (Rusek 1998; Waikhom et al. 2006; Gulvik 2007). Both groups respond relatively quickly to any changes in the soil environment and are commonly used as bioindicators of biological soil quality (Santofuro et al. 2012). Lehmann et al. (2010) claim that soil arthropods might be affected by biochar in relation to change in microorganisms’ biomass. Bunting and Lundberg (1987) found that springtails could feed on charcoal, but it is unclear whether it is beneficial to those organisms. Physical soil properties modified by biochar may also impact soil mesofauna. Large internal soil surface areas and pores are important for biological processes (Tisdall and Oades 1982). Improved bulk density and soil porosity may create better habitats for soil mesofauna (Twardowski et al. 2016). Also, the increase of pH as well as the improvement of water regime may directly affect soil microarthropod activity and abundance (Van Straalen and Verhoef 1997; Bardgett 2005).

The most widely used tests for biochar risk assessment are ecotoxicological bioassays, in particular of the well-studied springtail species *Folsomia candida* (Collembola: Isotomidae). Such tests have been successfully used with different biochars and doses on invertebrates (Domene et al. 2007; Domene et al. 2015a, b; Marks et al. 2014, 2016). The impact of biochar on springtails and mites, as the most abundant mesofauna representatives, within open-field conditions, fundamental for a better understanding of biochar application in practice is, however, relatively little studied. To diminish this knowledge gap, we conducted the study to determine the impact of biochar on soil mesofauna (primarily mites and springtails) in both laboratory and field conditions. The aim of our research was to evaluate the environmental risk of biochar used as a soil amendment to soil mesofauna (mites and springtails) in poor agricultural soil.

## Materials and methods

### Experimental design

The field experiment was set up in mid-April 2014 in the suburbs of Opole, Poland (50.5740 N, 17.8908 E) on poor (sandy and weakly acidic) agricultural soils and continued until October 2016. The climate of the area is moderately warm, with an average annual temperature of 8.4 °C and an average annual rainfall of 611 mm. The selected plants were maize and oilseed rape on account of these being common agricultural crops in Europe. Within each crop two treatments were compared: biochar and no biochar as a control, three replicates each. Each experimental plot was 3 × 3 m.

Before the start of the experiment the area was conventionally used as an agricultural field. The forecrop was maize. Pine chip biochar was applied up to a depth of 30 cm at a rate of 50 t/ha and was mixed by plowing, followed by sowing maize and oilseed rape seeds. In the field trials, no pesticides were used. Weeds were harvested manually a few times during the vegetation season. The maize variety was P8745 (FAO 250, Pioneer Company) and oilseed variety Monolit. The fertilizer in oilseed rape was ammonium sulfate 34% in a dose of 300 kg/ha, and in maize ammonium phosphate (Polydap) in a dose of 25 kg/ha. The same amount of fertilizers was applied in biochar and control treatments.

### Analysis of biochar and soil properties

The biochar used in the experiment was the product of low-temperature flash pyrolysis (300 °C) of pine and spruce chips. It is an industrial biochar produced by Fluid Company. The heating value of the tested biochar was 25 MJ/kg. The production had the capacity of 24 m^3^ of the plant chips/h, which is equivalent to the product in up to 2 MG of biochar/h. Before application, selected properties of biochar (pH, organic carbon content, cation exchange capacity, and heavy metal content) were analyzed according to International Biochar Initiative (IBI) Standard Product Definition and Product Testing Guidelines (IBI 2014; Table 1). The particle size fraction of biochar applied on the field was more than 2 mm (sieve method).

**Table 1:** The chemical characteristic of biochar used in the experiment

	Measured values	Limit values^a^
pH H_2_O	8.2	–
CEC (cmol/kg)	30.53	–
Carbon content (% of DM)	52.3	> 50
H/C_org_ ratio	0.026	< 0.7
NH_4_^+^ (mg/l)	0.04	–
NO_3_^−^ (mg/l)	0.08	–
Na^+^ (cmol/kg)	2.09	–
K^+^ (cmol/kg)	5.10	–
Mg^2+^ (cmol/kg)	1.80	–
Ca^2+^ (cmol/kg)	30.5	–
Pb (g/t DM)	1.57	< 150
Mn (g/t DM)	29.7	
Cu (g/t DM)	0.50	< 100
Hg (g/t DM)	0.32	< 1
Zn (g/t DM)	13.04	< 400
Cd (g/t DM)	2.0	< 40
Total PAH (sum of 16 EPAs PAH) (g/t DM)	< 0.001	< 6
${\mathrm{Pb}}_{{\mathrm{H}}_20}$ (g/t DM)	< 0.001	–
${\mathrm{Mn}}_{{\mathrm{H}}_20}$ (g/t DM)	7.63	–
${\mathrm{Cu}}_{{\mathrm{H}}_20}$ (g/t DM)	0.05	–
${\mathrm{H}\mathrm{g}}_{{\mathrm{H}}_20}$ (g/t DM)	–	–
${\mathrm{Zn}}_{{\mathrm{H}}_20}$ (g/t DM)	3.5	–

^a^According to IBI (2014)

Three times a year, the main soil physicochemical properties were measured (pH, organic carbon content, cation exchange capacity). Five samples were taken from each plot from a depth of 10 cm. Acidity (pH) in deionized H_2_O (*w*/*v*) was measured with a pH meter in soil samples in a standard dilution of 1:5 and in biochar in a modified dilution of 1:20 after 24 h equilibration on a shaker, according to Rajkovich et al. (2011). Cation exchange capacity (CEC) of control and biochar-amended soil was estimated from the equation between 1 M KCl exchangeable acidity and exchangeable Ca^2+^, Mg^2+^, Na^+^, and K^+^ measured at pH 7 with 1 M ammonium acetate (Reeuwijk 2002) on an MP 4200 Agilent spectrometer. Soil sorption capacity was estimated from the equation between 1 M KCl exchangeable acidity and CEC measured at pH 7 with 1 M ammonium acetate (Reeuwijk 2002) and compared biochar-amended soil with results from control soil.

The cation exchange capacity of biochar was calculated as a sum of Ca^2+^, Mg^2+^, Na^+^, K^+^, and NH_4_^+^ (Singh et al. 2017). Total organic carbon content was measured by dry combustion on a CHNS analyzer, CE Instruments. Total contents and bioavailable forms of Pb, Zn, Cu, Cd, Ni, and Mn in soil and biochar were measured on an MP 4200 Agilent spectrometer after sample digestion in 70% HNO_3_ (1:10 ratio) in a microwave system, Start D Milestone Instruments (EPA 2012) for total amounts or after extraction with distilled water in a 1:40 ratio (shaken for 2 h). Mercury content was measured in dry samples on an Hg analyzer MA-2, Nippon Instruments Corporation (NIC). Total PAH content in biochar was measured on HPLC Varian Co.

### Soil mesofauna field and laboratory studies

#### Field studies

Soil samples for mesofauna analysis were taken from all experimental parcels during plant growth. Each year soil was taken at a frequency of 1 month on three dates from May to July. During the soil sampling in years 2015–2016, maize was in growth stages according to the BBCH scale: 10–15, 32–37, and 61–67 and oilseed rape in stages 60–69, 72–79, and 83–89. The samples were taken with the use of a soil sampler (diameter 5 cm and depth 10 cm). The volume of one sample was 196 cm^2^. Soil invertebrates were extracted over 24 h from the soil samples with the use of Tullgren funnels modified by Murphy (1956). After the extraction, the springtails and mites were kept in 75% ethyl alcohol. Apart from springtails and mites, other mesofauna representatives occurred in the samples: mainly nematodes, proturans, spiders, and insect larvae. The mean abundance of these groups in samples was less than 5%.

#### Laboratory studies

For the avoidance and reproduction test we used the Collembola species *Folsomia candida* Willem 1902, which is one of the recommended springtail species for ecotoxicology tests. *F. candida* is a parthenogenetic, predominantly fungivorus species, living in the porous soil space International Standard Organization (ISO 1999a, b; Fountain and Hopkin 2005). The genus *Folsomia* was one of the species found in soil samples from the field experiment. The test species comes from a certified laboratory culture, obtained from the Institute of Industrial Organic Chemistry in Pszczyna, Poland. Before the test, the springtail was reared for 6 months on a plaster of Paris/charcoal substrate and fed with dry baker’s yeast. The culture was kept in a climatic chamber at a temperature of 21 °C, with the photoperiod 12 h light and 12 h dark. The culture was synchronized before the test.

The soil used in the tests was sampled from the control plots of oilseed rape and maize in early spring 2016. Before the test soil was sieved to 5 mm. Biochar was sieved to 2 mm and mixed with soil. The homogeneous volume, soil from oilseed rape or maize crop, was prepared in the following concentrations: 1, 1.5, 2.5, 5, 10, 25, and 50%. The analyzed concentrations correspond to doses of biochar per ha from 4.8 to 404 t (calculated on the basis of weight on biochar applicate on the field, and the specific gravity of the soil from experimental plots). The dose 50 t/ha in the experiment corresponds to the volume—percentage dose 10.0% (1.88% determined by weight). For each concentration, as well as for the control soil, the water holding capacity (WHC) as well as the pH were determined. Two days before the analysis, the moisture content of each sample was optimized to 50% of maximum WHC. Moistening was carried out with deionized water.

#### Avoidance test setup

The avoidance test was carried out in accordance with the (ISO 1999a. The test was carried out in six replications for each concentration of soil/biochar concentration. In total, 84 test vessels were prepared, 42 for the soil from oilseed rape and 42 for the soil from the maize field. The plastic test vessels (9 cm length × 7 cm width × 3 cm height) were filled, half with the control soil and half with the soil/biochar concentration (in a 30- to 30-g proportion, in order to maintain the same soil level on both sites of the glass) and tightly separated with a flat piece of glass. Subsequently, the glass separating the treatments from the control was removed and 20 individuals of *F. candida*, 10–12 days old, were carefully transferred to the center of the container. The containers were covered with transparent plastic film perforated with small holes (in order to allow air access) and left for 48 h at a temperature of 21 °C and the photoperiod 12 h light and 12 h dark. After this time, each soil portion from each site of the vessels was flooded with water with dark ink. The soil was thoroughly mixed with the water. After a few minutes, individuals were counted. Springtails on the water surface were counted as alive.

#### Reproduction test setup

The reproduction test was carried out following ISO 11267 (ISO 1999b). For each concentration, five replications were prepared, while for the control eight replications. In total, 50 plastic vessels were prepared for the soil from maize and 50 for the soil from the oilseed rape field. In order to control the soil moisture and pH during the test, one additional vessel from each concentration and control was prepared. The test vessels (6 cm diameter and 7.5 cm high) were filled with 30 g of control soil and the same volume of the tested concentrations. Twelve individuals of 10–12-day-old springtails were carefully transferred to each of the containers. At the beginning of the test, and at weekly intervals, springtails were fed with 2 mg of dry baker’s yeast. The containers were covered with transparent plastic film. The vessels were kept for 28 days at a temperature of 21 °C and the photoperiod 12 h light and 12 h dark. Each week, the plastic film was removed in order to aerate the test vessels. If necessary, water was supplemented in order to maintain 40–50% WHC. After 4 weeks, the test vessels were flooded with water and a small amount of ink and photographed to count the adults and juveniles on the water surface. Individuals were counted from photographs in the graphic program (ToupView software).

### Statistical analyses

The soil properties in maize and oilseed rape were compared with repeated ANOVA measures in the Agricolae Package in Software R (*p* ≤ 0.05). For the analyses of springtail and mite abundance the General Linear Model (GLM) was used in Statistica software, version 13.0. The qualitative factor was the experimental trial (biochar vs. control), while the quantitative factors were the year, sampling period (linked to the cropping season) and replication. The results were presented as the mean from 2 years, separately for maize and the oilseed rape crop. The effect of avoidance (*A*) for biochar concentrations was calculated using Eq. (1).

1 $$ A=\left(C=T\right)/N\Big)\times 100 $$

where *C* is the number of individuals in the control soil, *T* is the number of individuals in the test soil, and *N* is the total number of individuals

The number of individuals found in each concentration was compared with control (ANOVA, at significance level *p* ≤ 0.05). Additionally, both for the avoidance and reproduction tests, the dose-response model were used. The concentrations causing 50% effect (EC_50_) were determined from the dose-response curve, with nonlinear estimation and further least squares model estimation. The estimated function was *y* = *b*_0_ − *b*_0_ / (1 + (*x*/*b*_2_)**b_1_), where *b*_0_ was the expected response, *b*_2_ is the concentration for a half-maximal response, and *b*_1_ determines the slope of the function. These analyses were made in Statistica software, version 13.0.

## Results

### Biochar and soil properties

The tested biochar was alkaline—pH 8.2 (Table 1) and had 52.3% of carbon. The surface area of the tested biochar was low, only 16.5 m^2^/g, and cation exchange capacity was also lower − 39.5 cmol/kg, compared with biochars produced at higher temperatures and from other feedstock, like wheat straw, miscanthus, rice husk, or sewage sludge (Liang et al. 2006, 2010). It was free from PAH, and the concentration of all tested toxic compounds was very low or even under the level of detection, passing fixed recommendations for acceptable levels (IBI 2014).

Soil was collected only from the first 10 cm of cultivated A horizon and classified as Cambisol (FAO-WRB 2014), with typical sandy loam texture with the addition of medium gravel and pH_H2O_ from 7.1 to 7.3, which is higher than average for Polish soils, probably due to liming done in the past. Biochar significantly increased the organic soil carbon but only in the maize crop (*p* = 0.002) (Table 2). Conversely, biochar decreased soil pH, whose pH was significantly higher in the control (7.22) in comparison with trials, where biochar was applied (6.49), also only in maize. The application of biochar significantly increased the values of CEC in both trials (Table 2), due to the increase of exchangeable Ca^2+^, Mg^2+^, and H^+^ + Al^3+^ (exchangeable acidity) in the soil sorption complex.

**Table 2:** The soil conditions in maize and oilseed rape crop in trials where biochar was applied and control field

	Biochar	Control	F	p
Maize				
C_org_ (%)	0.94	0.78	16.30	0.002^a^
pH H_2_O	6.49	7.22	5.47	0.04
Na^+^ (cmol/kg)	0.12	0.18	7.80	0.02
Mg^2+^ (cmol/kg)	2.76	0.86	18.57	0.001
K^+^ (cmol/kg)	0.34	0.31	10.83	0.006
Ca^2+^ (cmol/kg)	4.05	2.28	3.92	0.07
Exchangeable acidity (cmol/kg)	1.71	1.1	4.21	0.08
CEC (cmol/kg)	8.98	4.73	6.28	0.03
Oilseed rape				
C_org_ (%)	0.94	0.92	0.112	0.75
pH H_2_O	6.88	7.26	0.000	0.99
Na^+^ (cmol/kg)	0.20	0.12	8.92	0.02
Mg^2+^ (cmol/kg)	2.14	1.13	12.96	0.007
K^+^ (cmol/kg)	0.30	0.19	22.87	0.001
Ca^2+^ (cmol/kg)	4.12	2.29	6.28	0.03
Exchangeable acidity (cmol/kg)	2.0	1.1	4.56	0.07
CEC (cmol/kg)	8.76	4.84	5.21	0.05

^a^Effect of treatment (results of repeated ANOVA)

The analysis of soil and biochar properties in the soil was linked with the experiments in laboratory conditions. The pH increased from 6.54 in natural soil to 8.46 in the 50% soil-biochar mixture. The water holding capacity increased by more than 3.5 times, comparing the natural soil and 50% soil-biochar mixture (Table 3).

**Table 3:** Soil pH measured in KCl and maximum water holding capacity (WHC) of soil–biochar concentrations in particular concentrations

Dose (%)	Maize		Oilseed rape	
	pH in KCl	WHC_max_ (%)	pH in KCl	WHC_max_ (%)
0.0	6.54	20.88	6.20	22.36
1.0	6.80	26.22	6.71	27.42
1.5	6.99	27.24	6.86	28.96
2.5	7.25	30.31	7.44	31.59
5.0	7.53	34.20	7.61	36.40
10.0	7.64	53.50	7.73	58.45
25.0	8.23	65.35	8.32	67.87
50.0	8.46	75.06	8.51	81.42

### Field studies on mesofauna

#### Maize

In the maize field, the mean abundance of three Acari suborders: Prostigmata, Gamasida, and Oribatida were significantly higher in the soil with biochar in comparison with the control (*p* = 0.0006, 0.04, and 0.08, respectively) (Table 4). In addition, the mean number of all mites (Acari all) was significantly higher in biochar in comparison with the control (*p* = 0.007). The mean number of Collembola in biochar treatment was not significantly different from the untreated one. The Collembola/Acari ratio was significantly higher in the soil with biochar, indicating better biological soil quality (*p* = 0.01).

**Table 4:** The mean abundance and ecological indices of mites and springtails in trials where biochar was applied and control in maize field

	Biochar	Control	F*	*p*
Prostigmata	10.94	3.5	11.95	0.0006
Astigmata	3.78	4.7	0.27	0.60
Gamasida	4.02	2.62	8.38	0.04
Oribatida	8.05	1.71	3.12	0.08
Acari (all)	21.59	12.5	7.42	0.007
Collembola (all)	4.32	4.60	0.03	0.85
Coll/Acari ratio	7.0	3.0	6.32	0.012

*F* and *p* are results of general linear model (GLM)

#### Oilseed rape

The mean number of mites from Prostigmata and Astigmata groups was significantly higher in biochar in comparison with the control (*p* = 0.018 and 0.035, respectively) (Table 5). Also, mites and springtails as whole communities were significantly more abundant in biochar in comparison with the control (*p* = 0.013 and 0.0006, respectively). The mean number of Collembola species, as well as the Collembola/Acari ratio, did not differ significantly.

**Table 5:** The mean abundance and ecological indices of mites and springtails in trials where biochar was applied and control in oilseed rape field

	Biochar	Control	*F*	*p*
Prostigmata	27.66	15.19	5.63	0.018
Astigmata	21.44	12.84	4.48	0.035
Gamasida	8.86	9.18	0.02	0.87
Oribatida	5.67	6.79	0.50	0.48
Acari (all)	65.09	42.57	6.18	0.013
Collembola (all)	27.59	8.14	12.01	0.0006
Coll/Acari ratio	1.45	1.09	0.77	0.38

*F* and *p* are results of general linear model (GLM)

### Laboratory experiment on springtails

#### Maize

**Fig. 1:**
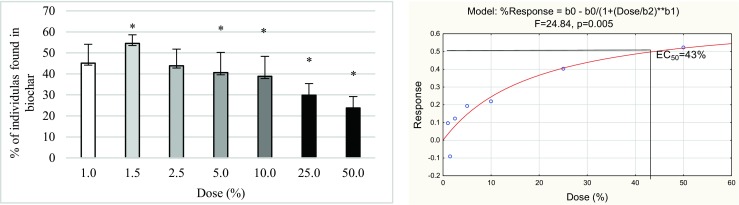
Avoidance behavior (**a**) and dose-response curve (**b**) of *F. candida* in all biochar concentrations mixed with soil from the maize field measured after 2 days of exposure. *p ≤ 0.05, significantly higher or lower value in comparison with control (ANOVA)

The mortality of *Folsomia candida*, both in the avoidance and reproduction tests, was less than 20% in all test vessels and did not differ significantly between treatments. In the avoidance test, doses from 5 to 50% significantly decreased the number of juveniles (Fig. 1).

**Fig. 2:**
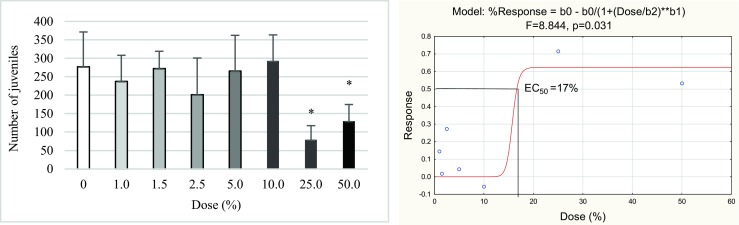
The mean number of juveniles (**a**) and the dose-response model of the reproduction test (**b**) found in seven biochar concentrations and control soil from the maize field after 28 days of incubation. *p ≤ 0.05, significantly higher or lower value in comparison with control (ANOVA)

At the same time, avoidance was not observed in the first three doses of biochar (1, 1.5, and 2.5%). The half maximal effective concentration (EC_50_) for this test was 43%. In the reproduction test, only the four last doses (from 25 to 50%) significantly inhibited the reproduction of Collembola (Fig. 2). The lower doses did not have any effect on the number of juveniles. The EC_50_ for the reproduction test was 17%.

#### Oilseed rape

**Fig. 3:**
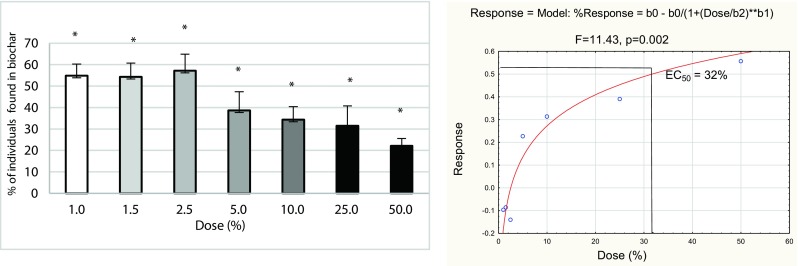
Avoidance behavior (**a**) and dose-response curve (**b**) of *F. candida* in all biochar concentrations mixed with soil from the oilseed rape field measured after 2 days of exposure. *p ≤ 0.05, significantly higher or lower value in comparison with control (ANOVA)

In all test vessels with soil from the oilseed rape crop, the mortality of *F. candida* was lower than 20%, both in the avoidance and reproduction tests. In the avoidance test, all doses had a significant impact (negative or positive) on *F. candida* (Fig. 3). The first three doses (from 1 to 2.5%) significantly increased the number of individuals in soil with biochar. From the 5.0% doses, avoidance was observed in the test vessels with biochar. The EC_50_ for the avoidance test was 32%.

**Fig. 4:**
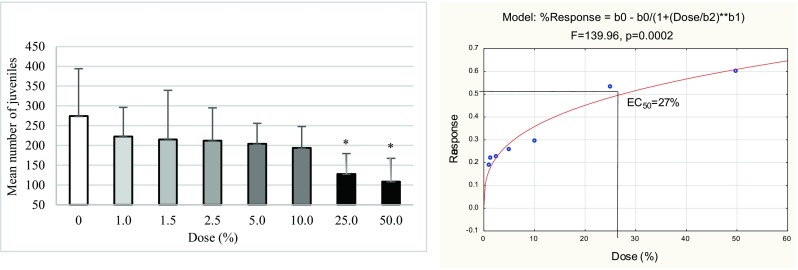
Mean number of juveniles (**a**) and the dose-response model of the reproduction test (**b**) found in seven biochar concentrations and control soil from the oilseed rape field after 28 days of incubation. *p ≤ 0.05, significantly higher or lower value in comparison with control (ANOVA)

In the reproduction test, only the last two doses (25 and 50%) negatively impacted *F. candida* reproduction (Fig. 4). The mean number of juveniles in other doses did not differ significantly between the particular biochar concentration and the control. EC_50_ for this test was 27%.

## Discussion

Our study is presumably the first attempt to study the interactions between biochar and soil-dwelling mesofauna (springtails and mites), simultaneously in field and laboratory conditions. The low-temperature pine/wood chip biochar used in the experiment had a high carbon content but low surface area and ability for nutrient storage, which was in agreement with the findings of other authors (Lee et al. 2016; Jiang et al. 2017). The analysis of metal concentration did not report any chemical interference by biochar amendment in the agro-ecosystem.

Potential impacts on soil biota and soil services related to biochar addition to soil can only be discussed at present, where specific biochar, soil type, and other experimental factors occur (Hilber et al. 2017). The application of biochar at the 50 t/ha rate in maize and oilseed rape crops significantly increased the mean number of mites and springtails, as well as the Acari/springtails ratio. The higher number of these organisms, as well as the higher value of ecological indices, shows the increase in soil biological quality in comparison with the control. Considering the results of other field experiments on biochar use in field conditions, no response of soil faunal feeding activity to biochar was found in the rate ranges from 0 to 30 t/ha (Domene et al. 2014). In rates from 12 to 48 t/ha, Zhang et al. (2013) found a significant increase of fungivorous nematode abundance and a decrease of plant parasites. Tammeorg et al. (2014) observed the positive response of earthworms to biochar (30 t/ha), but the result was not significant. Regarding epigeic arthropods, Castracani et al. (2015) did not find interaction between biochar and the abundance of epigeic macroarthropods in the rate of 14 t/ha. Concurrently with the findings of other authors (Neher and Barbercheck 1998; Gruss et al. 2018), we stipulate that the positive response of soil mesofauna to biochar addition was the result of improved physicochemical soil properties, in particular soil pH and organic matter content. In our research, the observed increase of organic carbon that followed biochar application is in agreement with a large number of previous observations (Lehmann 2007; Sohi et al. 2010; Xie et al. 2015; Vaccari et al. 2015). The liming effect of biochar was not observed under either of the tested plants. Acidity decrease after biochar addition can be explained by the release of organic acids and ammonia from material during microbial decomposition but can also be affected by crop cultivation. The addition of wood chip biochar significantly increased the sorption capacity of the tested soils, which was observed in higher cation exchange capacity and water content. These effects were also demonstrated in other field experiments (Conte et al. 2013; Baronti et al. 2014; Głąb et al. 2016; Yang et al. 2017). In our experiment, the increase in pH of an already alkaline soil did not influence the composition and abundance of target organisms, suggesting that this macroarthropod community is generally well adapted to alkaline environments.

In most of the bioassays on biochar on soil fauna, the concentrations are determined by weight. The biochar used for bioassays in our study had low particle size and great volume in small weight. Therefore, we determined volume-percentage concentrations. Our concentrations respond to the weight doses from 0.2 to 15%. The results of avoidance and reproduction tests were similar in the maize and oilseed rape crops. However, significant avoidance was noted at lower doses in comparison with the reproduction test.

In the short-term response, *F. candida* showed significant avoidance to biochar in doses from 5 to 50% (0.9 to 15% by weight). Furthermore, the 1.5% doses in maize and from 1 to 2.5% in oilseed rape significantly increased the number of *F. candida* in the test vessels with biochar. The reason might be the considerable increase in pH, from 6.54 (maize) and 6.20 oilseed rape to 8.46 and 8.51 in the dose of 50%. Van Straalen and Verhoef (1997) found that different mites and springtail species preferred soil pH varying from 2.9 to 7.6 tested in the maximum range 2 to 9. That means that these organisms preferred the more acid environment and avoided alkaline. Greenslade and Vaughan (2003) found that the highest rate of reproduction of *Folsomia candida* was observed in the range of pH form 3.5 to 6.6.

Domene et al. (2015b), after 2 days of incubation of *Folsomia candida*, found significant avoidance to maize stover biochar at a rate of 2%, which corresponds to 10% in our trials. In this case, biochar only slightly increased pH (form 7 to 7.5), but this probably caused the avoidance. Conti et al. (2017) found that the rates of different gasification biochars above 5% (in weight) caused the avoidance in *F. candida*. As the main reasons, authors report pH, PAH, and heavy metals. Li et al. (2011) found the avoidance of earthworm species *Eisenia fetida* to apple wood chip biochar in doses of 0.1 and 0.2%. While in this study traces of PAH were detected, the authors claim that avoidance might be the effect of insufficient moisture in soil with biochar (Li et al. 2011). In our tests, the soil moisture was controlled at 50% WHC. The relation between biochar contaminants and toxicity to soil faunal organisms often remains unexplained (Hilber et al. 2017).

In the reproduction test, we found a significant negative response to biochar from the rate 25% (5.0% by weight). In the study by Marks et al. (2014) biochars from pine feedstock obtained in pyrolysis and fast pyrolysis, as well as biochar obtained in poplar feedstock, positively affected the reproduction of *F. candida* in all doses. Domene et al. (2015b) did not find any significant effect of biochar obtained for different biomass sources (including pine wood) on *F. candida* reproduction in the rates from 0.2 to 14% (similar to our dose range). In our study, negative effects of biochar after 28 days of incubation were observed in relatively high concentrations, which are not applied in conventional agriculture.

## Conclusions

Soil mesofauna response to biochar was studied simultaneously in field and laboratory experiments. The analyzed biochar was obtained as a product of low-temperature flash pyrolysis (300 °C) of pine and spruce chips, and free from PAH, while the concentration of all tested toxic compounds was very low or even under the level of detection. In the field studies, biochar was applied on the field in the rate 50 t/ha in two crops—maize and oilseed rape. In laboratory tests (reproduction and avoidance test on *Folsomia candida*), the biochar risk assessment was conducted using soil from the field experiment. The dose 10% in the test corresponds to the dose 50 t/ha of biochar in the field studies. The application of the analyzed biochar in maize and oilseed rape crops significantly increased the mean number of mites and springtails. The Collembola/Acari ratio shows higher biological soil quality in the field where biochar was applied in comparison with the control. This positive effect probably resulted from improved soil chemical properties (in particular organic carbon content and cation exchange capacity) upon biochar addition. Considering the laboratory studies, in the short-term response (avoidance test), *Folsomia candida* showed a possible short-term toxicity risk from a dose of 5%. The results of the long-term response (reproduction test) indicate the negative response of *F. candida* from the rate of 25% (higher than the field dose, which corresponds to 10% in laboratory tests). It was concluded that the main reason for avoidance was the considerable increase in pH, which was also observed by other authors. Summarizing, the biochar used in the experiment positively affected soil invertebrates in the field studies, while in the laboratory studies, only short-term risk was observed. In would be interesting to research the impacts of biochars produced in different conditions on the same soils and organisms.
